# Comment on: Is clozapine use a risk of hematological malignancies? Insights from a meta‐analysis “Clozapine and Malignancy Risk”

**DOI:** 10.1111/pcn.13839

**Published:** 2025-05-10

**Authors:** Kazuki Nishida, Basile Chrétien

**Affiliations:** ^1^ Department of Biostatistics Kyoto University School of Public Health Kyoto Japan; ^2^ Department of International Medical Education Nagoya University Graduate School of Medicine Nagoya Japan; ^3^ Neuropresage Team, UNICAEN, INSERM, UMR‐S U1237, Physiopathology and Imaging of Neurological Disorders (PhIND), Institute Blood and Brain @ Caen‐Normandie (BB@C) Normandie University, GIP Cyceron Caen France

## Abstract

This article relates to Comment on: Is clozapine use a risk of hematological malignancies? Insights from a meta‐analysis “Clozapine and Malignancy Risk”.

We read with interest the recent research letter investigating the association between clozapine and hematological malignancies (HMs) through meta‐analysis.^1^ While the study raises an important pharmacovigilance issue and contributes to ongoing conversations about clozapine's safety, several critical methodological concerns need to be addressed before these findings can be reliably interpreted or used in clinical decision‐making. Ethics approval was not required for this commentary, as it does not involve human or animal participants or the analysis of original data.

One of the primary concerns with the meta‐analysis is the substantial heterogeneity among the studies included. The authors combined retrospective cohort studies, case‐control studies, and pharmacovigilance data, which introduces significant variability in study designs, patient populations, exposure definitions, and outcome measurements. It increases the risk of bias and makes it difficult to draw reliable conclusions.^2^ Although sensitivity and subgroup analyses were conducted, these do not fully address the fundamental differences between the studies. The reported *I* ^2^ statistic of 83% suggests high heterogeneity, and pooling data with this degree of variability undermines the strength and generalizability of the findings. Moreover, the authors did not mention adherence to the MOOSE (Meta‐analysis of Observational Studies in Epidemiology) guidelines for meta‐analyses of observational studies,^3^ nor did they provide information on protocol preregistration or use of the PRISMA (Preferred Reporting Items for Systematic Reviews and Meta‐Analyses) 2020 reporting framework.^4^ These elements are essential to ensure transparency and minimize the risk of selective reporting.

The approach to exposure measurement also presents limitations. The use of cumulative defined daily doses (DDDs) does not reflect treatment intensity, adherence, or pharmacokinetics. The chosen DDD cutoffs appear arbitrary and lack clinical justification. Without standardized exposure definitions and individual‐level data, it is difficult to make meaningful conclusions about the dose‐ or duration‐dependent risks of clozapine. The lack of harmonization across studies further complicates any attempt to interpret the findings in a clinically useful way.

The inclusion of different comparator groups among the studies also warrants concern. Some studies compared clozapine with other antipsychotics, others with olanzapine, and others with a nonclozapine‐treated population. These comparator groups differ in important ways, such as baseline risk, illness severity, and healthcare access, which introduces confounding.^5^ This complicates the interpretation of pooled effect estimates and raises questions about their validity. The authors do not sufficiently address how these differences were managed or accounted for. Without a more rigorous approach to selecting comparators, it is difficult to interpret the pooled results.

The inclusion of pharmacovigilance data from VigiBase^6^ in the quantitative analysis also raises concerns. While pharmacovigilance data can be useful for detecting early safety signals, they are not designed for estimating incidence rates or quantifying risks, as they are subject to reporting biases, lack denominators, and are often based on incomplete case verification. Including these data alongside cohort and case‐control studies introduces further heterogeneity and undermines the scientific robustness of the analysis. It would be more appropriate to treat pharmacovigilance data separately, using them to signal potential safety concerns rather than relying on them to contribute directly to a quantitative meta‐analysis.

Furthermore, the studies included in the analysis did not consistently adjust for important confounding factors such as smoking, obesity, comorbid conditions, or socioeconomic status—each of which is relevant both to the risk of hematological malignancies and to schizophrenia.^7^, ^8^ The use of the Newcastle‐Ottawa Scale to assess study quality is a reasonable approach, but this scale alone cannot ensure rigorous control of confounding. More advanced causal analysis methods would be necessary to more confidently assess the relationship between clozapine and HMs.

Finally, the interpretation of the findings should be tempered by the clinical context. Clozapine remains the most effective treatment for treatment‐resistant schizophrenia and is associated with a lower long‐term mortality rate compared to other antipsychotics.^9^ The absolute risk of HMs is low, and existing surveillance protocols are designed to monitor for potential hematological complications. Given these factors, the modest increase in HM risk observed in the meta‐analysis should not lead to undue alarm or discourage the appropriate use of clozapine. Clozaphobia has already contributed to its underuse, preventing many patients from benefiting from its unique therapeutic effects.^10^ Therefore, it is crucial that these findings are not misinterpreted in a way that could hinder its appropriate use.

In conclusion, while the study makes an important contribution to the ongoing investigation into clozapine's safety, the methodological weaknesses outlined here—such as high heterogeneity, issues with exposure definitions, questionable inclusion of pharmacovigilance data, and potential residual confounding—limit the strength of the conclusions that can be drawn. Further, more methodologically rigorous studies are needed to better understand the potential risks associated with clozapine and to clarify the true nature of any association between clozapine and hematological malignancies.

## Disclosure statement

The authors declare no conflicts of interest relevant to the content of this comment.

## Data Availability

No new data were generated or analyzed in support of this comment.
